# Tribe reassessment of the subhimalayan leafhopper genus *Pseudosubhimalus* (Homoptera: Cicadellidae) based on molecular phylogeny

**DOI:** 10.7717/peerj.7162

**Published:** 2019-08-28

**Authors:** GN Niranjana, Naresh M. Meshram, Pathour R. Shashank, Stuti, Tahseen Raza Hashmi

**Affiliations:** Division of Entomology, Indian Council of Agricultural Research-Indian Agricultural Research Institute, New Delhi, Delhi, India

**Keywords:** Phylogeny, Leafhopper, Tribe, Molecular, Deltocephalinae, Cicadellidae

## Abstract

The phylogeny of the *Pseudosubhimalus* were investigated using of two different data sets, including 91 taxa and 3853 aligned nucleotide positions from the histone *H3*, *28S* rDNA (D2 & D9–10 region). The results suggest the placement of genus in the tribe Ciacadulini, as it was clustered with Cicadulini genera. Relationships between genera of the Cicadulini were strongly supported and leads placement to tribe Cicadulini from Athysanini. Along with this, genus *Pseudosubhimalus* Ghauri is revised, and *P. trilobatus* sp. nov. (Himachal Pradesh: Katrain) is added, described from Indian subcontinent and deposited to National Pusa Collection, IARI, New Delhi, with repository number RRS1.

## Introduction

Genus *Pseudosubhimalus* are medium sized, ochraceous to dark brown with black spots on crown, distributed in Himalayan and Subhimalayan region. The species of genus were established by Ghauri (1974), with type species *Ophiola bicolor* Pruthi (Pruthi, 1936), as a replacement name for *Ophiola* Edwards. This genus was replaced to present name due to its general coloration which superficially resembles *Subhimalus* Ghauri (Ghauri, 1971). This genus was earlier placed in the tribe Deltocephalini (Oman, Knight & Nielson, 1990) of Deltocephalinae. Recently, Zahniser & Dietrich (2013), based on the molecular and morphological aspects of Deltocephalinae, placed it in the tribe Athysanini. *Pseudosubhimalus* is recorded from higher altitudes (8,000–12,000 ft), and mostly feed and breeds on grasses. This genus is distinguished from its related genera by aedeagal shaft with a pair of subapical processes; subgenital plates triangular, apophysis of style slender, tapering distally. Pruthi (1936) described the two species *P. bicolor* from India and *P. yatungensis* from Tibet.

In spite of the colour patterns and male genitalia character that characterize *Pseudosubhimalus*, the composition and placement of the genus have been problematic. Molecular data potentially provide numerous additional characters useful for phylogenetic hypotheses. Zahniser & Dietrich (2013) revised the classification of Deltocephalinae based on the molecular and morphological data and provided a revised interpretation of tribe Athysanini and placed this genus under this tribe. Here, we replaced *Pseudosubhimalus* to the tribe Cicadulini based on histone *H3*, *28S* rDNA (D2 & D9–10 region). Along with this morphological characterization of *Pseudosubhimalus* with a new species from India is provided.

## Material and Methods

### Collection of samples & morphological study

Collections were not done from any national park or other protected area of land or sea, or on any private land, hence no permission was required. No specific permissions were required for any of the collection localities/activities, as the collections were done in and around ICAR research institutes. The field studies did not involve any endangered or protected species. Specimens were collected through mercury vapour lamp light traps from different location in Himachal Pradesh, India were processed by a series of steps such as sorting, cleaning, and mounting. Male genitalia dissections were carried out as given by Oman (1949) and Knight (1965). The abdomen was removed by inserting a sharp pin between the abdomen and thorax and with gentle piercing. The abdomen will be treated in 10% KOH for 2–4 h to remove unsclerotized material by gently prodding the abdomen with the head of a pin. Afterwards, the abdomen was rinsed thoroughly in water. The internal structures were then removed by a hooked pin, before being stored in glycerol vials for study.

Photographs were taken with a Leica DFC 425C digital camera on the Leica M205FA stereozoom automontage microscope.

Type material is deposited in National Pusa Collection, IARI, New Delhi, with repository number RRS1.

**New Taxon LSID.** *Pseudosubhimalus*: urn: lsid:zoobank.org:act:766C2292-F762-4F2E-93A2-8745C84D7BCA, *Pseudosubhimalus trilobatus*: urn: lsid:zoobank.org:act:9A9DA3CD-A577-41DB-8059-28D57E38F875.

The electronic version of this article in Portable Document Format (PDF) will represent a published work according to the International Commission on Zoological Nomenclature (ICZN), and hence the new names contained in the electronic version are effectively published under that Code from the electronic edition alone. This published work and the nomenclatural acts it contains have been registered in ZooBank, the online registration system for the ICZN. The ZooBank LSIDs (Life Science Identifiers) can be resolved and the associated information viewed through any standard web browser by appending the LSID to the prefix http://zoobank.org/. The LSID for this publication is: urn:lsid:zoobank.org:pub:DB2CE2D9-1238-4199-96A4-A7FDCD98DD8B. The online version of this work is archived and available from the following digital repositories: PeerJ, PubMed Central and CLOCKSS.

### DNA extraction and PCR amplification

Total genomic DNA, from the legs of each specimens, were extracted with the help of the DNA Sure Tissue Mini Kit, following the manufacturer protocol. The extracted DNA was stored at −20 °C for further processing. The amplification of the desired product was done with the help diagnostic PCR reactions, using universal Histone H3 primers (HEX-AF 5′-ATGGCTCGTACCAAGCAGACGGC-3′ and HEX-AR 5′-ATATCCTTGGGCATGATGGTGAC-3′) (Ogden & Whiting, 2003) and 28S rDNA primers (for D2 region 5′-AGTCGKGTTGCTTGAKAGTGCAG-3′ & 5′-TTCGGGT CCCAACGTGTACG-3′) and for D9–D10 region 5′-GTAGCCAAATGCCTCGTCA-3′ & 5′-CACAATGATAGGAAGAGCC-3′ (Dietrich et al., 2001). The PCR protocol for Histone H3 followed Zahniser & Dietrich (2010) and 28S gene was amplified in 25 µl reactions using DNA polymerase (Fermentas GmBH, St. Leon- Rot, Germany) under the following cycling protocol: 4 min. hot start at 94 °C, 35 cycles of denaturation for 30 s at 94 °C, annealing for 60 s at 47 °C, elongation for 50 s at 72 °C and a final extension was carried out at 72 °C for 8 min in a C1000™ Thermal cycler (Meshram, Shashank & Sinha, 2017). The reactions were combined (as described by KOD FX puregene™ manufacturer protocol) of DNA template 4 µl, 2× PCR buffer 12.5 µl, 2 mM dNTP 10 µl, TAQ (KODFX) enzyme 1 unit, and forward and reverse primers were 0.3 µM each at final concentration. The products were checked on 1% agarose gel and visualized under UV using Alphaview® software version 1.2.0.1. The amplified products were sequenced at AgriGenome Pvt. Ltd. (Cochin, India). The quality sequences were assembled with BioEdit version 7.0.0 and deposited in NCBI GenBank (Table 1).

### Alignment and phylogenetic analyses

Sequences were aligned with the CLUSTAL W application in MEGA 6 (Tamura et al., 2013), the alignment was imported into BIOEDIT 7.0.9.0 (Hall, 1999), and minor changes were subsequently made by hand. NEXUS data block for combined analysis of histone *H3* & *28S* rDNA (D2 & D9–10 region), were prepared with following commands: #NEXUS begin data; dimensions ntax = 91 nchar = 3853; format datatype = dna interleave gap = −missing = N; matrix; end; for analysis through PAUP*4.0b10.

**Table 1:** GenBank accession numbers. A list of taxa included in the study and GenBank accession numbers.

**#S.No.**	**Species**	**Tribe**	**Accession number**	
			**28S**	**Histone H3**
1.	*Xestocephalus desertorum*	Aphrodinae	AF304619	GU123892
2.	*Acinopterus acuminatus*	Acinopterini	JX845484	GU123790
3.	*Acostemma* sp	Acinopterini	GU123696	GU123791
4.	*Acostemmini* gen. sp.	Acostemmini	JF835026	JN177306
5.	*Arrugada affinis*	Arrugadini	GU123699	GU123795
6.	*Atanus* sp	Athysanini	GU123700	GU123796
7.	*Brazosa picturella*	Athysanini	GU123709	GU123806
8.	*Cerrillus* sp	Athysanini	GU123711	GU123808
9.	*Chimaerotettix ochrescens*	Athysanini	JX845489	JX845530
10.	*Colladonus lineatus*	Athysanini	GU123718	GU123815
11.	*Dagama forcipata*	Athysanini	GU123720	GU123817
12.	*Euscelis seriphidii*	Athysanini	GU123729	GU123830
13.	*Eutettix pictus*	Athysanini	GU123730	GU123831
14.	*Eusama amanda*	Athysanini	AF304590	GU123829
15.	*Idioceromimus delector*	Athysanini	GU123740	GU123844
16.	*Loralia* sp	Athysanini	GU123746	GU123851
17.	*Napo* sp	Athysanini	GU123751	GU123856
18.	*Nesothamnus sanguineus*	Athysanini	GU123755	GU123860
19.	*Neohegira breviceps*	Athysanini	GU123753	GU123858
20.	*Neohegira* sp	Athysanini	GU123786	GU123891
21.	*Orientus* sp	Athysanini	GU123757	GU123862
22.	*Pachytettix* sp	Athysanini	GU123761	GU123865
23.	*Platymetopius obsoletus*	Athysanini	GU123771	GU123875
24.	*Renonus rubraviridis*	Athysanini	JX845524	JX845552
25.	*Thamnotettix confinis*	Athysanini	GU123783	GU123888
26.	*Twiningia* sp	Athysanini	GU123785	GU123890
27.	*Bahita* sp	Bahitini	GU123702	GU123798
28.	*Kinrentius* sp	Bahitini	JX845523	JX845549
29.	*Bonaspeia* sp	Bonaspeiini	JX845521	GU123804
30.	*Cerus goudanus*	Bonaspeiini	GU123712	GU123809
31.	*Renosteria waverena*	Bonaspeiini	GU123772	GU123878
32.	*Chiasmus* sp	Chiasmini	GU123713	GU123810
33.	*Gurawa minorcephala*	Chiasmini	X845495	JX856131
34.	*Listrophora styx*	Chiasmini	JX845500	JX845539
35.	*Nephotettix modulatus*	Chiasmini	GU123754	GU123859
36.	*Cicadula quadrinotata*	Cicadulini	GU123717	GU123813
37.	*Elymana acuma*	Cicadulini	GU123726	GU123826
38.	*Proceps acicularis*	Cicadulini	JX845511	JX845550
39.	*Cochlorhinus pluto*	Cochlorhinini	AF304586	GU123814
40.	*Deltocephalus* sp	Deltocephalini	GU123721	GU123819
41.	*Paramesodes* sp	Deltocephalini	GU123764	GU123868
42.	*Dorycephalus baeri*	Dorycephalini	JX845491	JX845532
43.	*Drabescus* sp	Drabescini	GU123724	GU123824
44.	*Bhatia satsumensis*	Drabescini	GU123706	GU123803
45.	*Drakensbergena retrospina*	Drakensbergenini	GU123725	GU123825
46.	*Eupelix cuspidata*	Eupelicini	AF304644	GU123828
47.	*Paradorydium lanceolatum*	Eupelicini	AF304637	GU123877
48.	*Hecullus bracteatus*	Faltalini	GU123737	GU123841
49.	*Tenucephalus* sp	Faltalini	GU123781	GU123886
50.	*Fieberiella florii*	Fieberiellini	AF304594	GU123834
51.	*Goniagnathus guttulinervis*	Goniagnathini	GU123736	GU123838
52.	*Glossocratus* sp	Hecalini	GU123735	GU123837
53.	*Attenuipyga vanduzeei*	Hecalini	AF304653	GU123822
54.	*Hecalus viridis*	Hecalini	AF304596	GU123840
55.	*Hypacostemma* sp	Hypacostemmini	GU123739	GU123843
56.	*Koebelia grossa*	Koebeliini	AF304599	GU123846
57.	*Limotettix striola*	Limotettigini	GU123745	GU123850
58.	*Balclutha neglecta*	Macrostelini	GU123704	GU123800
59.	*Dalbulus gelbus*	Macrostelini	AF304587	GU123818
60.	*Magnentius clavatus*	Magnentiini	JX845503	JX845541
61.	*Agrica arisana*	Mukariini	GU123779	GU123884
62.	*Mukaria maculata*	Mukariini	GU123750	GU123855
63.	*Occinirvana eborea*	Occinirvanini	JX845507	JX845545
64.	*Neoaliturus carbonarius*	Opsiini	GU123752	GU123857
65.	*Pseudophlepsius binotatus*	Opsiini	JX845512	JX845551
66.	*Hishimonus phycitis*	Opsiini	GU123738	GU123842
67.	*Japananus hyalinus*	Opsiini	JX845499	JX845538
68.	*Nesophrosyne maritima*	Opsiini	JX845506	JX845544
69.	*Opsius* sp	Opsiini	GU123756	GU123861
70.	*Orosius orientalis*	Opsiini	JX845509	JX845547
71.	*Aflexia rubranura*	Paralimnini	GU123698	GU123793
72.	*Laevicephalus monticola*	Paralimnini	GU123744	GU123849
73.	*Bandaromimus parvicauda*	Pendarini	GU123705	GU123802
74.	*Tropicanus chiapasus*	Pendarini	GU123784	GU123889
75.	*Jafar javeti*	Penthimiini	JX845498	JX845537
76.	*Penthimidia eximia*	Penthimiini	JX845510	JX845548
77.	*Penthimiola* sp	Penthimiini	GU123766	GU123871
78.	*Excultanus conus*	Phlepsiini	GU123732	GU123833
79.	*Phlepsius intricatus*	Phlepsiini	GU123768	GU123873
80.	*Anoplotettix fuscovenosus*	Scaphoideini	JX845486	JX845527
81.	*Scaphoideus* sp. n.	Scaphoideini	JX845513	JX845553
82.	*Scaphytopius frontalis*	Scaphytopiini	JX845514	JX845555
83.	*Adama elongata*	Selenocephalini	GU123694	GU123788
84.	*Selenocephalus* sp	Selenocephalini	GU123777	GU123881
85.	*Pachymetopius decoratus*	Stegelytrini	GU123760	GU123864
86.	*Kinonia elongata*	Stenometopiini	GU123741	GU123845
87.	*Stirellus catalinus*	Stenometopiini	AF304614	GU123882
88.	*Tetartostylus parabolatus*	Tetartostylini	GU123782	GU123887
89.	*Stymphalus rubrolineatus*	Vartini	GU123778	GU123883
90.	***Pseudosubhimalus bicolor***	Cicadulini	**(a) MK680069 (D2)**	**MH172175**
			**(b) MK680065 (D9–10)**	
91.	***Pseudosubhimalus trilobata*sp.nov.**	Cicadulini	**(a) MK680071 (D2)**	**MH172179**
			**(b) MK680067 (D9–10)**	

### Maximum Parsimony Bootstrap Analysis (MPBS)

Maximum parsimony (MP) analyses were run in PAUP*4.0b10 (Swofford, 1998) Analyses were run with the following search commands: ‘log file = mp-log; set autoclose = yes; set maxtrees = 100 increase = no; set criterion = parsimony; outgroup; hsearch addseq = random nreps = 100 multrees = yes hold = 1 swap = tbr; showtrees; describetrees 1/plot = phylogram brlens = yes; pscores ALL/tl = yes ci = yes ri = yes rc = yes hi = yes; savetrees file = mp-all.tre root = yes brlens = yes; bootstrap nreps = 1000 keepall = yes/AddSeq = random nreps = 100 savereps = yes; savetrees file = mp-boot.tre from = 1 to = 1 savebootp = both maxdec = 0 root = yes brlens = yes; pscores ALL/tl = yes ci = yes ri = yes rc = yes hi = yes; log stop’.

## Results

### Taxonomy

#### *Pseudosubhimalus* Ghauri

*Pseudosubhimalus* Ghauri, 1974: 553. Type species *Ophiola bicolor* Pruthi, 1936, by replacement.

**Diagnosis**. Male: Small leafhoppers, 3.0–4.0 mm long, dark brown to pale yellow. Crown, pronotum and scutellum marked with irregular dark brown spot. Pleurite black, spine on the legs pale yellow. Face black to ohraceous with irregular transeverse dark brown to yellow stripe. Forewings ochraceous to dark brown with hyaline venations with dark brown mottling.

**Figure 1:**
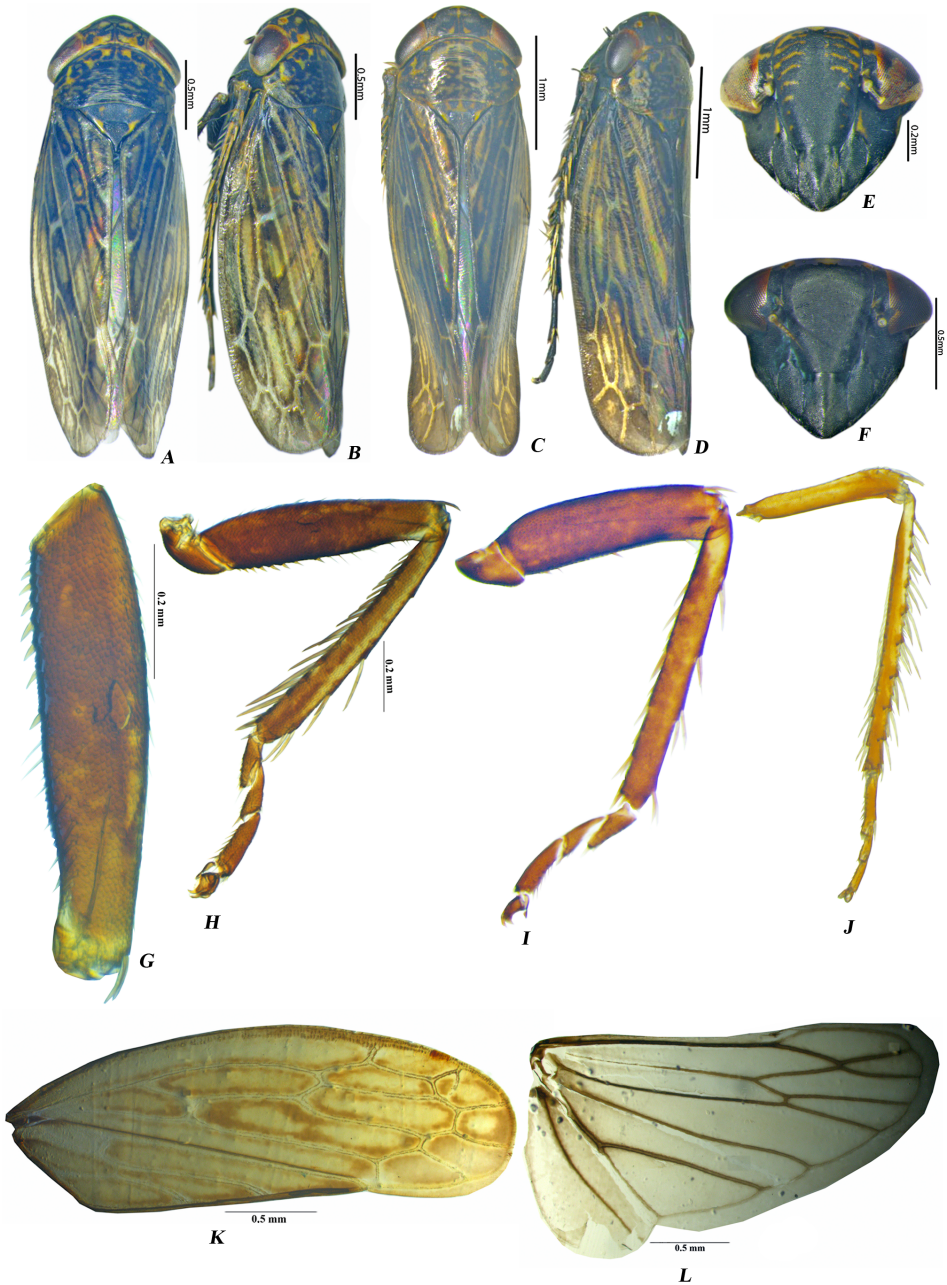
Male *Pseudosubhimalus* species (A) *P. bicolor* dorsal habitus. (B) *P. bicolor* lateral habitus*.* (C) *P. trilobatus* sp.nov. dorsal habitus. (D) *P. trilobatus* sp.nov. lateral habitus. (E) *P. bicolor* face. (F) *P. trilobatus* sp.nov. face. (G–L) *P. bicolor* male (G) Fore Femur (H) Fore leg (I) Middle leg (J) Hind leg (K) Forewing (L) Hind wing. Photo credit: Naresh M. Meshram.

**Description***.* Head conical to subconical, as broad as pronotum or broader, crown broad; frontoclypeus longer than wide, lateral margins broadly convex; clypeal sulcus distinct; clypellus elongate with sides parallel over basal 0.66, narrowed at apex, apically slightly exceeding the facial margin; genal margins concave beneath eyes; ocelli small, situated near anterior margin of crown by a distance equal to its own diameter, eyes large, occupying less than $ \frac{1}{2} $ of entire dorsal area of head. Pronotum short, median length about equal to median length of crown, almost three times as wide as its median length, surface knobbed, combined length of mesoscutum and scutellum longer than median length of pronotum, scutellum shorter than mesoscutum. Forewing (Fig. 1K) with distinct venation, with three anteapical cells, outer anteapical and middle cells closed; outer apical cell elongate, appendix narrowed not well developed. Hindwing venation complete with one anal vein; m-cu crossvein subapical (Fig. 1L). Prothoracic femur, with AV (anteroventral) stout setae present, AV1 setae long, hairlike, (Fig. 1G). Prothoracic tibia with dorsal surface rounded but not expanded (Fig. 1H); AD (anterodorsal) and PV (posteroventral) setae sparse; PD (posterodorsal) setae very dense; AV setae dense and long (Fig. 1H). Mesothoracic femur with sparse AV setae (Fig. 1I). Metathoracic femur with setal formula 2 + 1 + 1 (Fig. 1J); lateral surface broadened distally with dense setae distally (Fig. 1J). Metathoracic tibia flattened, tibial row PD with long macrosetae, AD with macrosetal bases spine like, AV with macrosetae, PV with numerous long tapered setae, tarsomere II less than 1/2 length of tarsomere I& II (Fig. 1J).

**Figure 2:**
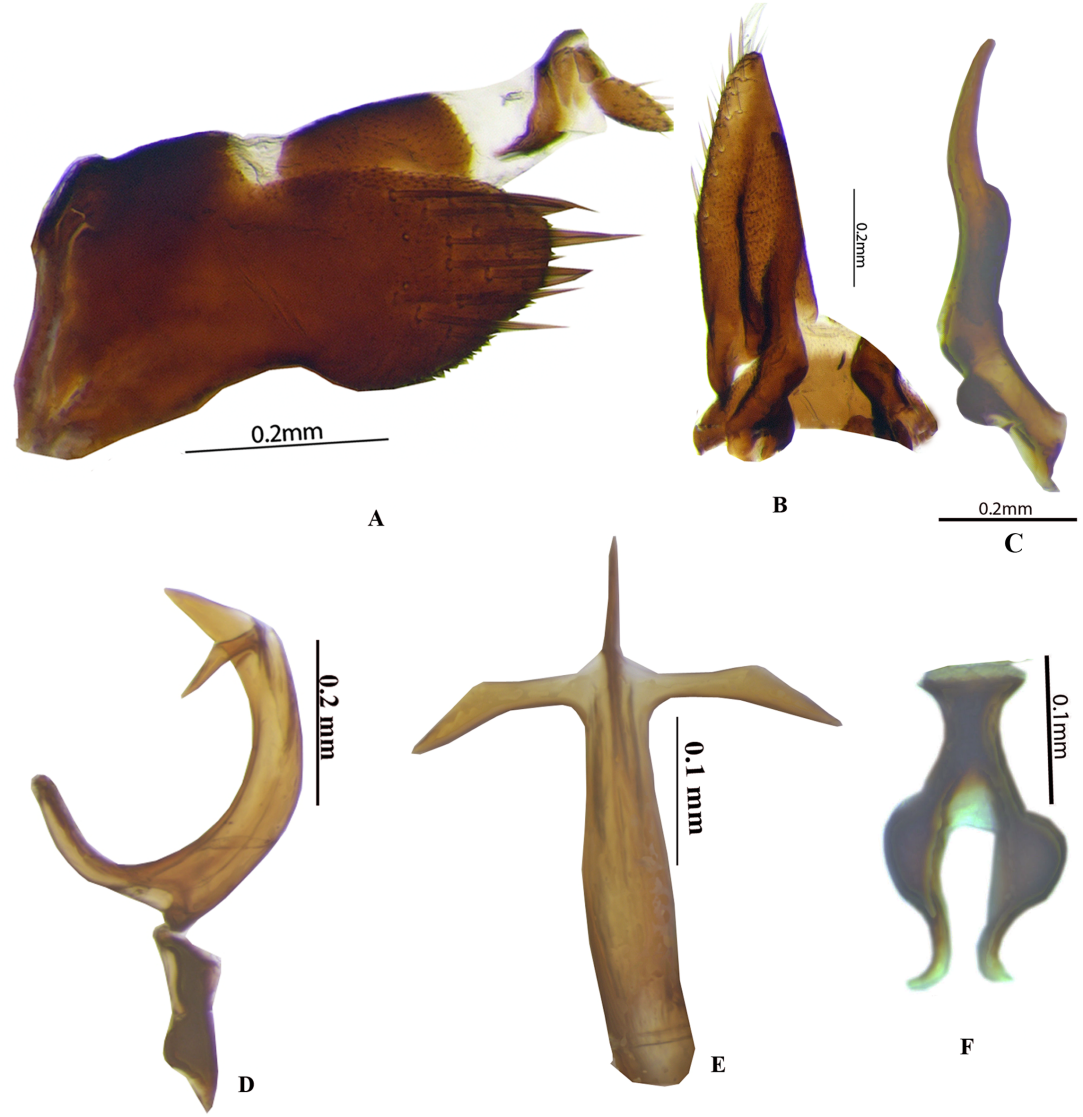
Genitalia male *P. bicolor* (Pruthi). (A) Pygofer lateral. (B) Subgenital plate with valve. (C) Style. (D) Aedeagus lateral. (E) Aedeagus ventral. (F) Connective. Photo credit: Stuti.

**Genitalia**. Male: Pygofer in lateral view (Fig. 2A) broad anteriorly and narrowed posteriorly, with long setae on posterior half, dorsal and ventral posterior margin with or without minute serrations. Anal tube elongate, long, well sclerotised. Subgenital plate (Fig. 2B) triangular in shape with wide base, sharply narrowed posteriorly, with long setae laterally and hair like long setae posteriorly. Connective (Fig. 2F) Y-shaped with stem shorter than arm. Style broad at base (Fig. 2C) with well-developed preapical lobe, apophysis long, slender, with blunt apex, >0.25 of total style length. Aedeagus with two or four processes, gonopore subapical.

**Figure 3:**
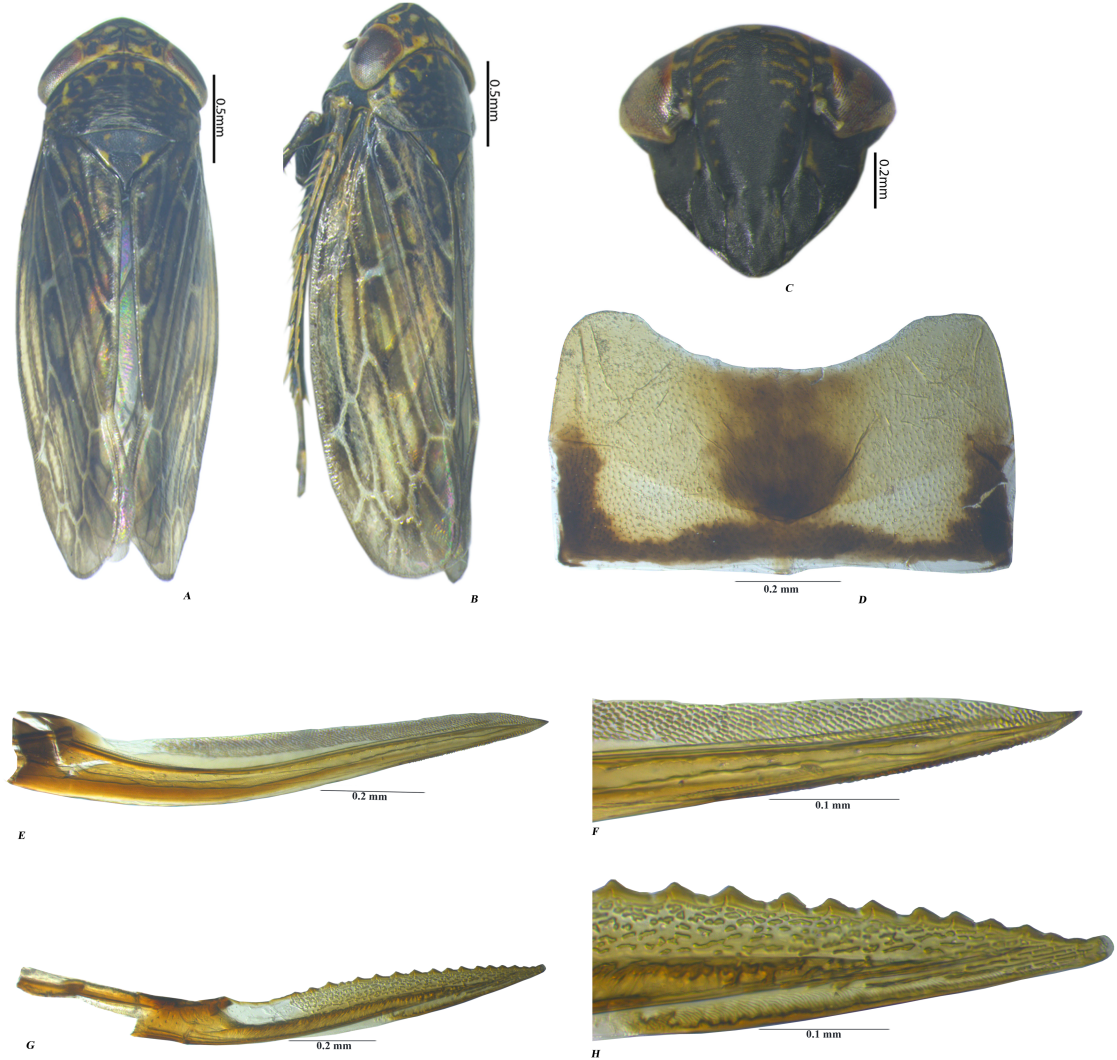
Female *P. bicolor* (Pruthi) (A–H). (A) Habitus dorsal. (B) Habitus lateral. (D) Seventh sternite. (E–F) Valvulae I (GH) Valvulae II. Photo credit: Naresh M. Meshram.

**Female** **genitalia.** Adult ♀: VII sternite hind margin broadly concave (Fig. 3D). Valvulae I (Figs. 3E–3F), in lateral view, with apex acute; dorsal sculptured area extending from base portion to apex of blade, formed mostly by scale-like processes arranged in oblique lines. Valvulae II (Figs. 3G–3H), in lateral view, moderately expanded beyond basal curvature; dorsal margin concave in the middle with dorsal hyaline area and convex apically on toothed areas, dorsal margin with 17 teeth with irregular reticulation on distal 1/3rd, dorsal margin.

### Key to species of *Pseudosubhimalus* (Males)

**Table:** 

1.	Pygofer ventral margin with dentations, aedeagus acute apically.............*P. bicolor* (Pruthi)
-	Pygofer ventral margin smooth.......................................................................................2
2.	Aedeagus bulbous at base with pair of very small subapical processes (Pruthi, 1936: Figs. 136a, b)..............................................................................................*P. yatungensis* (Pruthi)
-	Apex of aedeagus trilobed, subapical processes long (Figs. 4D and 4E).................... *P. trilobatus* sp. nov.

### *Pseudosubhimalus bicolor* (Pruthi) [Figs. 1A, 1B, 1E, 1G–1L, 2A–2F, 3A–3H]

**Table:** 

*Ophiola bicolor*Pruthi, 1936: 123
*Pseudosubhimalus bicolor* (Pruthi): Ghauri, 1974: 554

**Diagnosis:** Coloration, structure of the specimens studied agrees well with the description of the species by Pruthi (1936), except that the head is ochraceous, leg ochraceous. Male genitalia structure also shows some variations. Pygofer 1.4× longer than broad, with long setae on posterior half, dorsal and ventral posterior margin with minute serrations.

**Figure 4:**
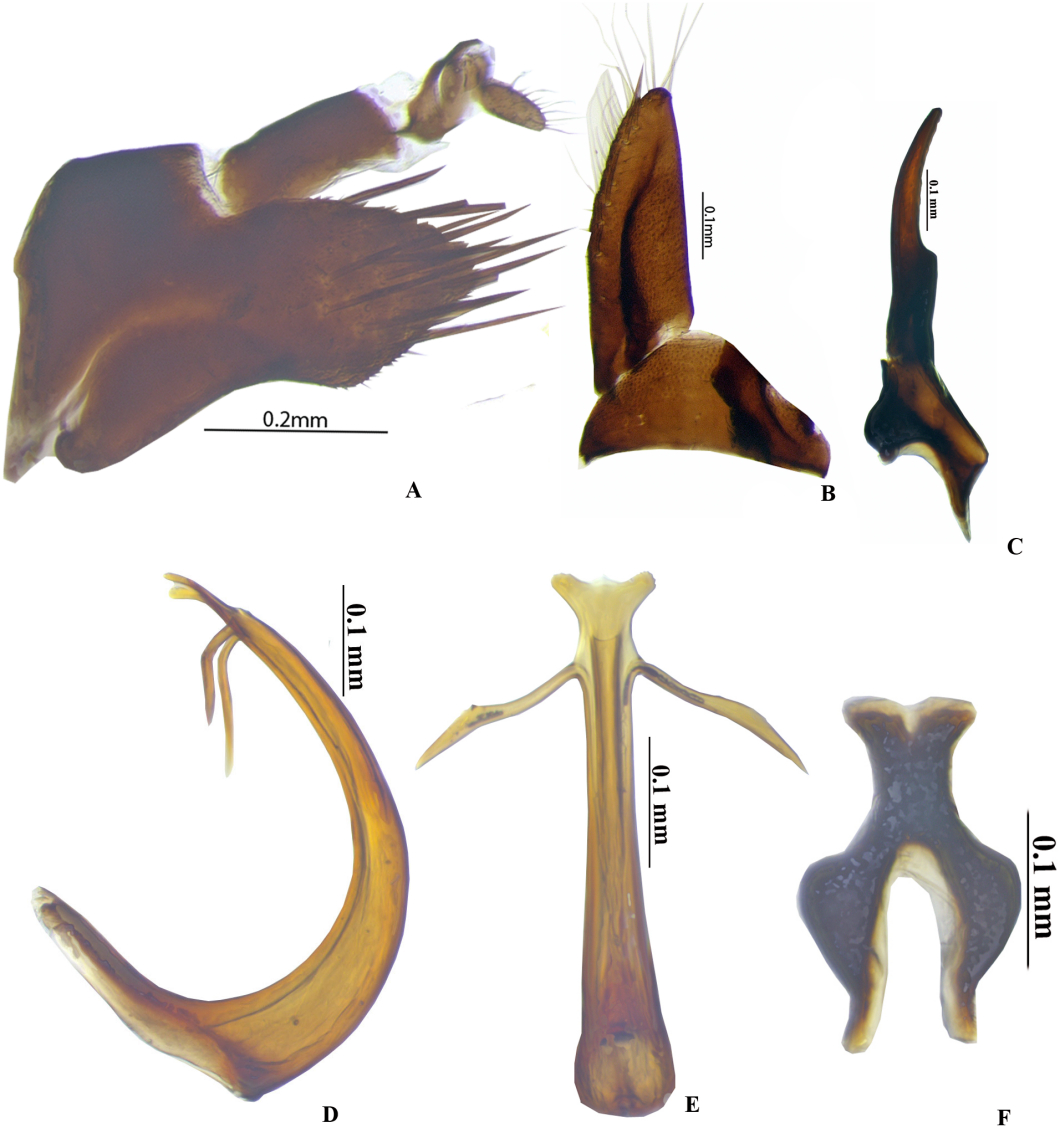
Genitalia male *P. trilobatus* sp. nov. (A) Pygofer lateral. (B) Subgenital plate with valve. (C) Style. (D) Aedeagus lateral. (E) Aedeagus ventral. (F) Connective. Photo credit: Niranjana GN.

**Description*.*** Male: Dark brown color, Crown, pronotum and scutellum marked with irregular dark brown spot. Face black to ochraceous with irregular transeverse dark brown stripe. Forewings dark brown with hyaline venations with dark brown mottling.

Head including eyes 1.09× width of pronotum (Fig. 1A), in dorsal view obliquely rounded in front, crown length 0.35× width across eyes; face length 0.9× width of face. Ocelli near anterior margin of crown and distance between eye and ocellus equal to diameter of ocellus. Pronotum 0.5× as long as width and 0.8× length of scutellum.

**Genitalia*.*** Male. Pygofer (Fig. 2A) is longer 1.4× than broad, with long setae on posterior half, dorsal and ventral posterior margin with minute serrations. Subgenital plate (Fig. 2B) triangular with wide base, sharply narrowed posteriorly, with setae 10 along lateral margin. Connective Y-shaped (Fig. 2F) with stem shorter 0.6× than arm. Style (Fig. 2C) broad at base with well-developed preapical lobe, apophysis long, slender, with blunt apex, 0.33 of total style length. Aedeagus in lateral view (Fig. 2E) narrowed basally, pointed apically and moderately broad medially with two small traingluar subapical processes. Gonopore subapical at the base of the processes.

**Female*.*** Adult ♀: Seventh sternite (Fig. 2D) hind margin broadly concave. Valvulae I (Figs. 2E–2F), in lateral view, with apex acute; dorsal sculptured area extending from base portion to apex of blade, formed mostly by scale-like processes arranged in oblique lines. Valvulae II (Figs. 2G–2H), in lateral view, moderately expanded beyond basal curvature; dorsal margin concave in the middle with dorsal hyaline area and convex apically on toothed areas, dorsal margin with 17 teeth, with irregular reticulation on distal 1/3rd, dorsal margin.

**Measurement** **(mm)**: Total Length: 7.1; Crown Length: 0.5; Width of Head: 1.9; Width of Pronotum: 2.4.

**Matrial examined**. INDIA: 11 ♂ & 15 ♀ Himachal Pradesh, Katrain, (76°59′N, 32°30′E and 3,300 msl) 23.ix.2016, sweep net Coll. Niranjan. Himachal Pradesh: Palchau, 11 ♂ & 8 ♀ 07.vi.1987, Coll. V.R.S. Rao (NPC); Uttarakhand: 11 ♂ & 8 ♀, Tehri Garhwal, 12.x.1988, Coll. V.V. Ramamurthy (NPC).

### *Pseudosubhimalus trilobatus* sp. nov. Meshram & Niranjana [Figs. 1C, 1D, 4A–4F]

**Diagnosis*.*** *P. trilobatus* sp. nov. resembles *P. bicolor* (Pruthi) in coloration and external morphology but can be distinguished by certain male genitalia characters like pygofer dorsal and ventral posterior margin without serrations. Aedeagal shaft narrowed apically, with trilobed apex in dorsal view. Gonopore subapical placed above base of the processes.

**Description*.*** Male: Dark brown color, Crown, pronotum and scutellum marked with irregular dark brown spot. Face completely black without any marking. Compound eyes black with reddish tinge and ocelli orange color. Forewings dark brown with hyaline venations with dark brown mottling.

Head including eyes 1.1× width of pronotum (Fig. 1C), in dorsal view obliquely rounded in front, crown length 0.35× width across eyes; face length 0.87× width of face. Ocelli near anterior margin of crown and distance between eye and ocellus equal to diameter of ocellus. Pronotum 0.5× as long as width and 0.8× length of scutellum.

**Genitalia*.*** Male: Pygofer is longer than broad (Fig. 4A), posterior margin conically rounded, macrosetae confined to posterior half, dorsal and ventral posterior margin without serrations. Valve broadly triangular (Fig. 4B), subgenital plate long (Fig. 4B), triangular uniseriate macrosetae and fringe of very long fine setae along. Connective Y-shaped (Fig. 4F); stem narrowed, 0.6× smaller than arms. Style broad basally (Fig. 4C), apophysis long, slender, with blunt apex, 0.33 of total style length. Aedeagus, in lateral view (Fig. 4D), broadly C-shaped, shaft narrowed apically, with trilobed apex in dorsal view, with two subapical processes, length of process is 0.33 of total length in dorsal view (Fig. 4E). Gonopore subapically placed above base of the processes.

**Measurement** **(mm).** Total Length: 7.1; Crown Length: 0.5; Width of Head: 1.9; Width of Pronotum: 2.4.

**Etymology*.*** Species name refers to the three lobed apex of aedeagus.

**Type** **Material*.*** Holotype: 1 ♂ INDIA: Himachal Pradesh, Dalang maidan, (32.6192°N, 77.3784°E and 3,300 msl) 24.ix.2016, sweep net Coll. Niranjan. Paratype 6 ♂ same data as on Holotype.

**Holotype Submitted to NPC-** Repository number: RRS1.

**New Taxon LSID.** *Pseudosubhimalus*: urn: lsid:zoobank.org:act:766C2292-F762-4F2E-93A2-8745C84D7BCA, *Pseudosubhimalus trilobatus*: urn: lsid:zoobank.org:act:9A9DA3CD-A577-41DB-8059-28D57E38F875.

**Figure 5:**
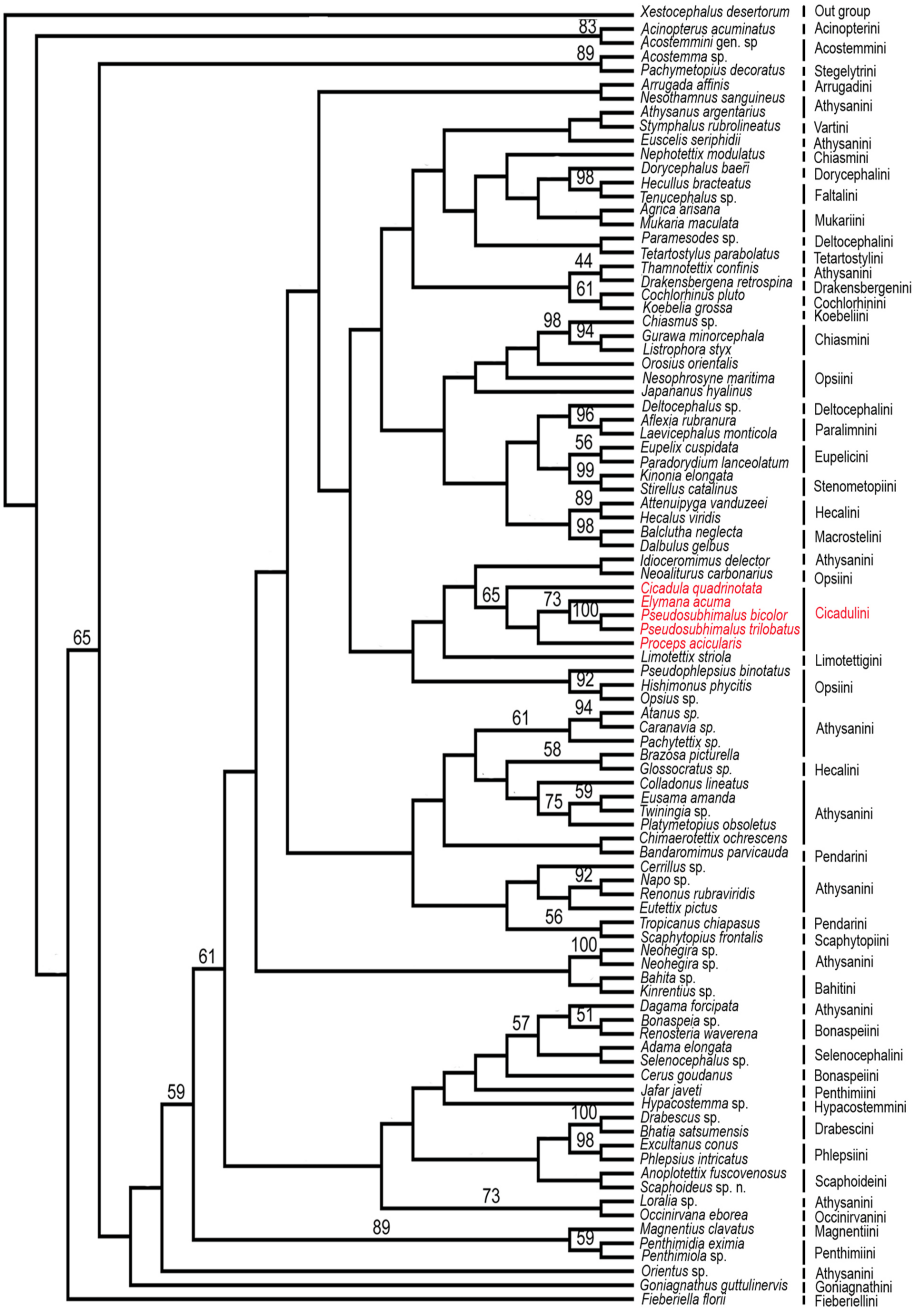
Strict consensus parsimonious tree resulting from combined parsimony analysis of histone *H3* and *28S* rDNA gene (D2 & D9–10 region).

### Molecular analysis

Maximum Parsimony Bootstrap analysis of the 91 taxa and 3853 aligned nucleotide positions from the histone *H3* and *28S* rDNA gene (D2 & D9–10 region) by PAUP*4.0b10 was done and yielded strict consensus tree (Fig. 5). The genus *Pseudosubhimalus*, which was sister to *Elymana* well-supported clade (>50 MPBS). This suggest the placement of the genus in the tribe Cicadulini. Our combined analysis resulted to be the most closely (*Cicadula*, *Proceps*, and *Elymana*), which resolved the placement of the genus to the tribe Ciacaulini from Atthysanini.

## Discussion

The systematic position of *Pseudosubhimalus* (Ghaurii), as suggested by morphological evidence, is ambiguous. This genus can be distinguished from its closely associated genera by the following combination of characters: 1. Prothoracic femur, with AV stout setae present, AV1 setae long, hairlike, (Fig. 1G). 2. Prothoracic tibia with dorsal surface rounded but not expanded (Fig. 1H); AD and PV setae sparse; PD setae very dense; AV setae dense and long (Fig. 1H). Mesothoracic femur with sparse AV setae (Fig. 1I). Metathoracic femur with setal formula 2 + 1 + 1(Fig. 1J); lateral surface broadened distally with dense setae distally (Fig. 1J). Metathoracic tibia flattened, tibial row PD with long macrosetae, AD with macrosetal bases spine like, AV with macrosetae, PV with numerous long tapered setae, tarsomere II less than 1/2 length of tarsomere I & II (Fig. 1J).

In *Pseudosubhimalus* anal tube elongate, long, well sclerotized which strongly suggest the placement of this genus to tribe Cicadulini. To confirm actual phylogenetic position of the *Pseudosubhimalus* in the context of the subfamily Deltocephalinae, we performed a preliminary molecular analysis (Maximum Parsimony Bootstrap analysis) by using available material of a series of taxa belonging to different tribes of Deltocephalinae from NCBI GenBank (Table 1). Our molecular results exclude *Pseudosubhimalus* from a close relationship among the genera of the previously placed tribe Athysanini (Zahniser & Dietrich, 2013) and placed it in the tribe Cicadulini. Our histone *H3*, *28S* rDNA (D2 & D9–10 region) bases combined analysis resulted to be the most closely (*Cicadula*, *Proceps* and *Elymana*) or the most distantly related (*Xestocephalus desertorum*) (Fig. 5). The final data matrix of our preliminary phylogenetic analysis (Table 1) included 91 terminals (90 ingroup taxa of Deltocephalinae and 1 outgroup taxon). Overall, these results improved our understanding of the systematic position of *Pseudosubhimalus.*

## Conclusion

Previously *Pseudosubhimalus* placed within the tribe Athysanini and our combined gene phylogenetic analysis leads its placement to the tribe Cicadulini. Present study reassessed the taxonomic position of genus *Pseudosubhimalus* with a more robust and well-resolved phylogeny. Which will help ongoing evolutionary and taxonomic work. Our molecular analysis based on Maximum Parsimony Bootstrap Analysis (PAUP) leads the placement of the *Pseudosubhimalus* to the tribe Cicadulini from Athysanini based on histone *H3*, *28S* rDNA (D2 & D9–10 region). Overall, these results improved our understanding of the systematic position of *Pseudosubhimalus*; however, more rigorous evaluations with larger numbers of genes are still necessary in the future.

## Additional Information and Declarations

### DNA Deposition

The following information was supplied regarding the deposition of DNA sequences: Data is available at GenBank, accession numbers: MH172175, MH172179, MH172183 and MH172181.

### Data Availability

The following information was supplied regarding data availability: Type material is deposited in National Pusa Collection, IARI, New Delhi, with repository number RRS1.

### New Species Registration

The following information was supplied regarding the registration of a newly described species: Publication LSID: urn:lsid:zoobank.org:pub:DB2CE2D9-1238-4199-96A4-A7FDCD98DD8B. *Pseudosubhimalus* LSID: urn:lsid:zoobank.org:act:766C2292-F762-4F2E-93A2-8745C84D7BCA. *Pseudosubhimalus trilobatus* LSID: urn:lsid:zoobank.org:act:9A9DA3CD-A577-41DB-8059-28D57E38F875.

## References

[ref-1] Dietrich CH, Rakitov RA, Holmes JL, Black WC (2001). Phylogeny of the major lineages of membracoidea (Insecta: Hemiptera: Cicadomorpha) based on 28S rDNA sequences. Molecular Phylogenetics and Evolution.

[ref-2] Ghauri MSK (1971). A new genus of Euscelinae from the Lower Himalayas, and a new species of *Balclutha* Kirkaldy (Homoptera, Cicadelloidea). Bulletin of Entomological Research.

[ref-3] Ghauri MSK (1974). New genera and species of Cicadelloidea (Homoptera, Auchenorrhyncha) from economic plants in India. Bulletin of Entomological Research.

[ref-4] Hall TA (1999). BioEdit: a user-friendly biological sequence alignment editor and analysis program for Windows 95/98/NT. Nucleic Acids Symposium Series.

[ref-5] Knight WJ (1965). Techniques for use in the identification of leafhoppers (Homoptera: Cicadellidae). Entomologist’s Gazette.

[ref-6] Meshram NM, Shashank PR, Sinha T (2017). A new genus of leafhopper subtribe Paraboloponina (Hemiptera: Cicadellidae) with molecular phylogeny of related genera. PLOS ONE.

[ref-7] Ogden TH, Whiting M (2003). The problem with the Paleoptera problem: sense and sensitivity. Cladistics.

[ref-8] Oman PW (1949). The Nearctic leafhoppers (Homoptera: Cicadellidae). A generic classification and checklist. Memoirs of the Entomological Society of Washington.

[ref-9] Oman PW, Knight WJ, Nielson MW (1990). Leafhoppers (Cicadellidae): a bibliography, generic check-list, and index to the world literature 1956–1985.

[ref-10] Pruthi HS (1936). Studies on Indian Jassidae (Homoptera). Part III. Descriptions of some new genera and species, with first records of some known species from India. Memoirs of the Indian Museum.

[ref-11] Swofford DL (1998). PAUP* Phylogenetic analysis using parsimony (*and other methods) version 4.0b10.

[ref-12] Tamura K, Stecher G, Peterson D, Filipski A, Kumar S (2013). MEGA6: molecular evolutionary genetics analysis version 6.0. Molecular Biology and Evolution.

[ref-13] Zahniser JN, Dietrich CH (2010). Phylogeny of the leafhopper subfamily Deltocephalinae (Insecta: Auchenorrhyncha: Cicadellidae) based on molecular and morphological data with a revised family-group classification. Systematic Entomology.

[ref-14] Zahniser JN, Dietrich CH (2013). A review of the tribes of Deltocephalinae (Hemiptera: Auchenorryncha: Cicadellidae). European Journal of Taxonomy.

